# Evaluation of a Smartphone Decision-Support Tool for Diarrheal Disease Management in a Resource-Limited Setting

**DOI:** 10.1371/journal.pntd.0005290

**Published:** 2017-01-19

**Authors:** Farhana Haque, Robyn L. Ball, Selina Khatun, Mujaddeed Ahmed, Saraswati Kache, Mohammod Jobayer Chisti, Shafiqul Alam Sarker, Stace D. Maples, Dane Pieri, Teja Vardhan Korrapati, Clea Sarnquist, Nancy Federspiel, Muhammad Waliur Rahman, Jason R. Andrews, Mahmudur Rahman, Eric Jorge Nelson

**Affiliations:** 1 Institute of Epidemiology, Disease Control, and Research (IEDCR), Bangladesh Ministry of Health and Family Welfare, Dhaka, Bangladesh; 2 Infectious Diseases Division (IDD), International Centre for Diarrhoeal Disease Research, Bangladesh (icddr,b), Dhaka, Bangladesh; 3 Quantitative Sciences Unit, Stanford University School of Medicine, Stanford, California, United States of America; 4 Department of Pediatrics, Stanford University School of Medicine, Stanford, California, United States of America; 5 Nutrition and Clinical Services Division, International Centre for Diarrhoeal Disease Research, Bangladesh (icddr,b), Dhaka, Bangladesh; 6 Geospatial Center, Stanford University Libraries, Stanford, California, United States of America; 7 Independent Technology Developer, San Francisco, California, United States of America; 8 BeeHyv Software Solutions Pvt. Ltd., Hyderabad, India; 9 Department of Medicine, Stanford University School of Medicine, Stanford, California, United States of America; Johns Hopkins Bloomberg School of Public Health, UNITED STATES

## Abstract

The emergence of mobile technology offers new opportunities to improve clinical guideline adherence in resource-limited settings. We conducted a clinical pilot study in rural Bangladesh to evaluate the impact of a smartphone adaptation of the World Health Organization (WHO) diarrheal disease management guidelines, including a modality for age-based weight estimation. Software development was guided by end-user input and evaluated in a resource-limited district and sub-district hospital during the fall 2015 cholera season; both hospitals lacked scales which necessitated weight estimation. The study consisted of a 6 week pre-intervention and 6 week intervention period with a 10-day post-discharge follow-up. Standard of care was maintained throughout the study with the exception that admitting clinicians used the tool during the intervention. Inclusion criteria were patients two months of age and older with uncomplicated diarrheal disease. The primary outcome was adherence to guidelines for prescriptions of intravenous (IV) fluids, antibiotics and zinc. A total of 841 patients were enrolled (325 pre-intervention; 516 intervention). During the intervention, the proportion of prescriptions for IV fluids decreased at the district and sub-district hospitals (both *p* < 0.001) with risk ratios (RRs) of 0.5 and 0.2, respectively. However, when IV fluids were prescribed, the volume better adhered to recommendations. The proportion of prescriptions for the recommended antibiotic azithromycin increased (*p* < 0.001 district; *p* = 0.035 sub-district) with RRs of 6.9 (district) and 1.6 (sub-district) while prescriptions for other antibiotics decreased; zinc adherence increased. Limitations included an absence of a concurrent control group and no independent dehydration assessment during the pre-intervention. Despite limitations, opportunities were identified to improve clinical care, including better assessment, weight estimation, and fluid/ antibiotic selection. These findings demonstrate that a smartphone-based tool can improve guideline adherence. This study should serve as a catalyst for a randomized controlled trial to expand on the findings and address limitations.

## Author Summary

Diarrheal disease is responsible for one in ten deaths among children less than five years of age globally. Innovative interventions to address gaps in the clinical care of these patients are lacking, yet will likely reduce the morbidity and mortality from diarrheal diseases. Therefore, the objective of this pilot study was to take a technology-enabled approach to improve guideline adherence, including antibiotic selection for diarrheal disease management in a resource-limited setting. To do this we adapted WHO guidelines to a smartphone platform and evaluated the approach in Bangladesh at two rural hospitals. The platform was durable and demonstrated positive improvement in guideline adherence. The results suggest that the decision-support tool was associated with a decrease in intravenous fluid use while maintaining safety, an increase in use of the recommended antibiotic, and a decrease in use of medications not recommended. This study represents a critical first step towards technology-enabled decision-support tools for diarrheal disease in resource-limited settings.

## Introduction

The provision of high-quality clinical care in resource-limited settings is challenged by logistical, educational, and temporal constraints that are exacerbated by a high volume of patients. This is especially true for the management of diarrheal diseases, which may overwhelm health facilities amid outbreaks or seasonal swells of disease. These diseases disproportionately burden poor communities and remain the second leading cause of death for children less than 5 years of age [1–3]. While outpatient decision-support tools like the paper-based World Health Organization (WHO) Integrated Management of Childhood Illness (IMCI) have improved community efforts despite limitations [4–8], inpatient references like the WHO Pocketbook of Hospital Care for Children are often scarce or anecdotally don’t meet providers needs in high-volume situations [9].

These collective challenges manifest in poor guideline adherence and exacerbate the ongoing struggle to improve care for patients with diarrheal disease, especially during large-scale outbreaks. For example, weight-based dosing is fundamental to pediatric care, however equations to estimate weight are rarely both accurate and user-friendly/accessible at the bedside [10–13]. Secondly, the inappropriate use of antibiotics is driving the proliferation of drug resistant pathogens; however prescription behavior change is challenging with complex unpredictable barriers, especially in developing countries [14]. Lastly, complications from basic interventions, such as fluid resuscitation, are more nuanced in resource-limited settings than previously thought [15, 16]. Relatively high rates of post-discharge mortality suggest that improved in-hospital efforts might decrease overall morbidity and mortality [17].

This pilot study aimed to address a subset of these challenges by adapting the WHO diarrhea management guidelines onto a smartphone platform and evaluating the approach in resource-limited hospitals. A simple ‘Rehydration Calculator’ was developed based on end-user feedback; functionality included weight-for-age estimation, a dehydration assessment guide, and treatment recommendations. The calculator was evaluated in a clinical study at two remote hospitals in Bangladesh to determine how technology-enabled decision-support impacts standard of care for the management of diarrheal disease.

## Methods

### Ethics Statement

This pilot study was approved by the Institutional Review Boards (IRB) at the IEDCR (IEDCR/IRB/2015/03) and Stanford University School of Medicine (6208). Written informed consent was obtained from all adult participants and guardians of minors (< 18 years); assent was obtained for children 11–17 years of age. The human experimentation guidelines of the US Department of Health and Human Services were followed during the conduct of this research.

### Technology Development

**Fig 1:**
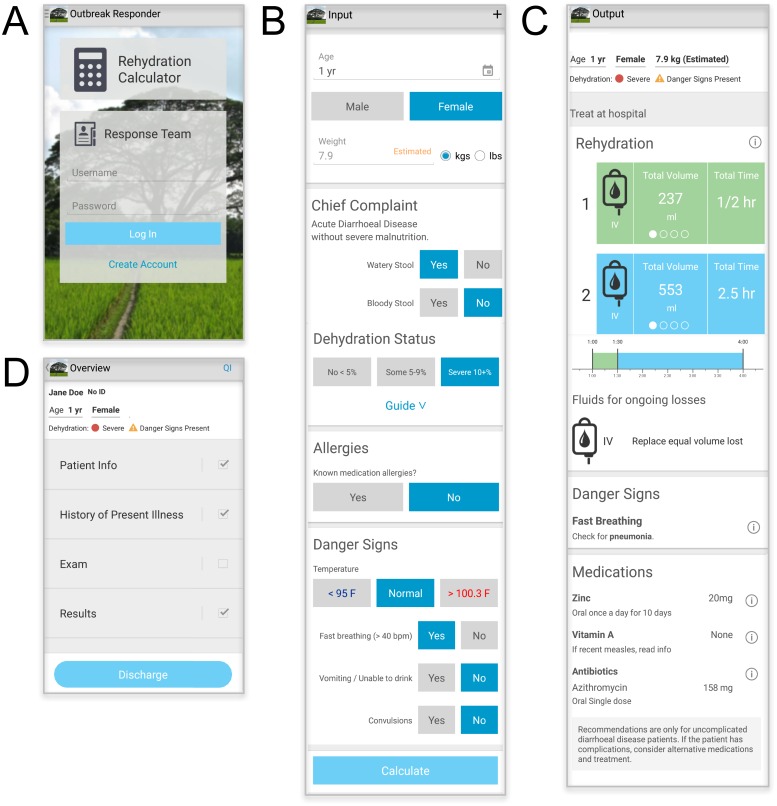
Rehydration calculator. (A) Home page with the Rehydration Calculator for decision-support (upper) and the data collection platform (lower). (B) Data input page. (C) WHO derived recommendations for medication and fluid administration; units can be changed by side-swiping. “i” represents a link to educational summaries and primary publications. (D) Outbreak Responder data collection and aggregation platform for the research team (aka ‘Response Team’). ‘QI’ is a portal for customized quality improvement questions intended to be used by researchers only.

Two software modalities were developed and utilized in this study (Fig 1A; S1 Movie; S1 Software). First: the WHO-guidelines were adapted onto a smartphone for decision-support and coined the ‘Rehydration Calculator’ (Fig 1B and 1C). Its development relied on end-user guidance (e.g. field clinical providers) to make it fast, desired, and durable in high-volume, low-resource settings. The calculator captures no personal health information and is not password protected. The calculator does, however, capture basic information to calculate the recommended treatment plan (aka age, gender, watery/bloody stool, five clinical signs of dehydration, allergies, and danger signs). On the back-end, these symptoms are encrypted, stamped with the time and GPS location, and can be aggregated for syndrome-based surveillance. Second: a wireless data collection and aggregation platform (aka the Outbreak Responder platform) was built for the research team (aka ‘response team’). It was developed based on end-user guidance (e.g. field research staff and epidemiologists) to be durable in settings with limited connectivity, yet capture essential epidemiologic data. The user interface is organized as a medical chart and collectively captures critical demographic, syndromic, laboratory, and outcomes information. The sections are ‘Patient Information’, ‘History of Present Illness,’ ‘Exam’, and ‘Results’ (Fig 1D). The Outbreak Responder captures personal health information and is therefore, password protected, encrypted, and built to industry standards for health information data security. Both technologies are Android-based, function on/off line, and are available upon request (S1 Software). Although the software was built and tested using a locally produced low-cost smartphone (Walton, Bangladesh; 80 USD), a Samsung Note 3 device (200–400 USD) was used in this study because of the improved battery life, ideal screen size (5 in.), and screen responsiveness.

### Enrollment and Study Design

#### Location

This pilot study was conducted in the rural northern district of Netrokona, Bangladesh (population of 2.2 million). Netrokona was chosen because it was remote, extremely resource-limited, and has frequent diarrheal disease outbreaks, including cholera [18]. There is one district and ten sub-district government hospitals in Netrokona that collectively receive approximately 5000 patients with diarrheal disease per month; the study was conducted at the main district hospital and at one sub-district hospital (Madan) by the Bangladesh government’s Institute of Epidemiology, Disease Control and Research (IEDCR) in collaboration with Stanford University.

#### Timeline

The study consisted of a 6 week pre-intervention and 6 week intervention period; standard of care was maintained throughout the study except that the Rehydration Calculator was used by the admitting clinician during the intervention period only. The study was conducted from September to December 2015, when cholera outbreaks typically occur in northern Bangladesh [19]. The pre-intervention and intervention periods each spanned 36 study days from September 19 to November 5, 2015, and from November 15 to December 29, 2015, respectively. Prior to both the pre-intervention and intervention periods, onboarding periods were required from September 5 to September 18, 2015 and from November 6 to November 14, 2015, respectively. During this time, additional individualized training was provided (see implementation and training section below) to the research staff prior to the pre-intervention and to admitting clinicians prior to the intervention. These onboarding periods also helped mitigate a possible Hawthorne effect from the presence of the research staff (pre-intervention) or presence of the Android device (intervention) at admission.

#### Inclusion criteria

Inclusion criteria were patients two months of age and older presenting with diarrheal disease (three or more loose stools per 24 hours) at the district hospital’s diarrhea ward or the sub-district hospital’s diarrhea corner [17, 20]. During the pre-intervention period, patients with co-morbidities were excluded, with the exception of severe malnutrition, which allowed for minimal disruption of the standard of care. During the intervention period, patients with clinical signs of severe malnutrition were also excluded because severely malnourished patients require highly detailed clinical management that was beyond the capacity of the two sites and the current design of the calculator. Patients with severe malnutrition were referred to a pediatric sub-specialist or to a tertiary care facility.

#### Primary outcome

The primary outcome was adherence to WHO guidelines for diarrheal disease management with and without the decision-support from the Rehydration Calculator. Increased adherence was defined as a statistically significant increase in orders of recommended treatment during intervention compared to pre-intervention (e.g. IV fluid use and volume ordered, use and type of antibiotic ordered, use of zinc).

#### Implementation

Before the pre-intervention period, approximately 100 clinicians and medical staff received didactic training in the respective hospital conference rooms (2 hours). The curriculum included an epidemiologic review of diarrheal disease in the study area and explanation of the study goals and design [to observe standard of care with and without the use of the Rehydration Calculator]. It was emphasized that the study design was planned to minimize disruption. A refresher on rehydration guidelines was not performed and paper-based decision-support was not provided. Physicians were told to assess dehydration per their training; in general, this region had been trained to use the Dhaka Method (icddr,b method) during past cholera outbreaks (see clinical assessment and management section). It was required that the physicians record onto the admission sheet the physician’s name, patient name, patient age/ gender, diarrhea type (e.g. watery, bloody), dehydration assessment (no, some, severe), and treatment plan (fluids and medications). The role of the research staff was limited to data and sample collection; a separate multi-day training was provided to the research staff on how to perform consent/ assent, collect clinical data, collect samples, and transfer data from the paper record to the Outbreak Responder software.

Prior to the intervention, a second training was conducted. Clinicians were trained on how to use the Rehydration Calculator in a didactic format with trainees using test phones (2 hours). The curriculum consisted of a review of the study design and goals. Materials included diagrams highlighting the use of the Rehydration Calculator by the admitting physician. The clinicians were trained to assess and generate the treatment recommendations with the Rehydration Calculator and to transcribe recommendations from the Rehydration Calculator onto the admission sheet. Clinicians were free to deviate from the recommendations based on situational concern.

One smartphone (Samsung Note 3) was allocated to each field research assistant and medical technologist. One shared smartphone was provided to each admitting group of doctors. When the clinical phone needed to be charged, the phone was exchanged for a charged phone by the research staff. Additional individualized clinician training was provided as needed during the onboarding periods; the need for additional training was directed towards users with no prior smartphone experience. Data collection and recording in the intervention was conducted in the same manner as the pre-intervention. Monitoring and evaluation were conducted under the guidance of a research study coordinator/ physician: calls were placed daily to research staff and senior clinical representatives of the study sites, onsite evaluation was performed every 2–3 weeks, and review of the hard and soft copy registry was conducted at the study midpoint and endpoint. The onsite evaluation consisted of group feedback sessions to identify any clinical or study concerns.

### Clinical Assessment and Management

#### Weight estimation

Many resource-limited health care facilities lack scales. When faced with this situation in Bangladesh, the standard of practice is to use a ‘best guess’ to estimate weight-for-age. The 2011 and 2014 Bangladesh Demographic and Health Survey reported that the average healthy pediatric weight in the Dhaka Division (contains Netrokona District) is at the 2006 WHO -1 weight for age z-score [21]. For children between 2 months and less than 10 years of age, the calculator was therefore designed to make sex-specific weight estimates to the WHO -1 z-score [22]. For children 10 to less than 15 years of age, sex-specific weight estimates were set to the USA Centers for Disease Control (CDC) -1 z-score [23] since WHO weight-for-age data are not available for these ages. For ages 15 to less than 20 years of age, estimates were set to 42 kg (females) and 45 kg (males). For patients 20 years of age and older, estimates were set to 45 kg (females) and 50 kg (males); these estimates were based on weight measurements collected during the pre-intervention period. In order to maintain consistency with the guidelines and standard of care, no correction was made based on dehydration status.

#### Clinical assessment

During the pre-intervention period, clinicians classified dehydration status by standard of care, independent of the calculator. However during the intervention period, the clinician used the Rehydration Calculator to gather essential data and to calculate the treatment plan based on the WHO-derived rehydration guideline called the Dhaka Method (Fig 1) [9, 13]. Although there are multiple assessment methods with different strengths [24, 25], the Dhaka Method was chosen because the local staff were most familiar with this modality. Dehydration status was classified into ‘none’, ‘some’, and ‘severe’ dehydration which approximates 0–4%, 5–9%, and 10% weight loss, respectively. The calculator used a Bangladesh Ministry of Health and Family Welfare/ icddr,b adjustment to the WHO algorithm for dehydration assessment [9, 13]. In brief, ‘some’ dehydration requires at least two findings of restlessness/irritability, sunken eyes, drinks eagerly or thirsty, and the skin pinch goes back slowly (two to less than three seconds) with at least one *key* finding being restlessness/irritability or drinks eagerly/thirsty. ‘Severe’ dehydration occurs when the criteria for ‘some’ dehydration are met plus at least one key finding in the highest category of lethargy/unconscious, skin goes back very slowly (three or more seconds), drinks poorly/unable to drink, or uncountable/absent peripheral pulse. No dehydration (‘none’) is the default when neither criteria for ‘some’ nor ‘severe’ are met.

#### Clinical management

During the pre-intervention period, the clinicians provided standard of care without use of the Rehydration Calculator; however, during the intervention period, the clinicians used the Rehydration Calculator to assess and calculate the WHO fluid and medication recommendations [4, 9, 26]. In brief, severely dehydrated patients (10% fluid loss by body weight) were recommended to receive intravenous fluid resuscitation of a total volume of 100 ml per kg; the time course of administration was age dependent [9]. Azithromycin was recommended for severely dehydrated patients with acute watery diarrhea, which was based on antibiotic sensitivity patterns at the icddr,b [27–29]. Given that the study was conducted in a cholera endemic region during the fall period when cholera outbreaks typically occur, azithromycin was also recommended for patients with some dehydration (5–9% fluid loss by body weight) from acute watery diarrhea [30]. Based on icddr,b and WHO recommendations, the calculator recommended pivmecillinam [31, 32] for patients with bloody diarrhea, suggestive of shigellosis. The calculator recommended zinc for all patients less than 5 years of age; although zinc supplementation will not cause harm, it was not recommended for patients older than 5 years of age [9].

### Microbiological Assays

A subset of stool samples (first and last patient per day) underwent targeted culture and sensitivity testing for Vibrio cholerae, *Salmonella* spp. and *Shigella* spp. [33] in the IEDCR and the International Centre for Diarrhoeal Diseases, Bangladesh (icddr,b) laboratories; stool swabs were placed in Cary Blair media, stored at four degrees Celsius, and transported from the remote field site on a bimonthly to monthly basis. Assays for additional pathogens (e.g. ETEC, cryptosporidium, norovirus, rotavirus) were not performed because the intent of the cultures was to identify antibiotic sensitivity patterns for pathogens that clinically warrant antibiotic treatment, not for comprehensive surveillance.

### Data Collection for Hospital and Post-Discharge Course

Basic secondary outcomes were collected from the discharge record (e.g. discharge type, mortality). At 10-days post-discharge, the research staff called the patient/guardian to collect secondary outcomes (e.g. readmission, mortality). Data were aggregated and reported by the research staff via the Outbreak Responder platform; the post-discharge calls were also placed from within the software.

### Statistical Analysis

Patient characteristics were described by percentages for dichotomous (yes/no) variables and by the median, 1^st^ and 3^rd^ quartiles (Q1, Q3) for continuous data. For dichotomous variables, significant differences were assessed by the two-sided Fisher’s Exact test at the 0.05 level; the risk ratio (RR) was calculated by dividing the proportion of individuals with the event during the intervention period by the proportion of individuals with the event during the pre-intervention period; thus, an RR < 1 indicates the event is less likely to occur in the intervention and an RR > 1 indicates the event is more likely to occur in the intervention. For continuous variables, significance at the 0.05 level was assessed by either the two-sided Wilcoxon signed rank test (comparison to a median) or the two-sided Wilcoxon rank sum test. When calculating the weight-adjusted IV fluid volume and the recommended IV fluid volume, measured weights were used where available, and otherwise (i.e., sub-district intervention), estimated weights were used. To calculate congruence during the intervention between IV fluid volume ordered and the recommended IV fluid volume, we used the Wilcoxon rank sum test to test if volume ordered was more than 30% different than what was recommended.

Standard of care at both study hospitals relies on a best guess weight estimation without a measured weight, however weight was independently measured to evaluate the weight estimation performed by the calculator. To assess how well estimated weights matched measured weights for children younger than 15 years of age, we calculated the difference between estimated and measured weight and used the Wilcoxon signed rank test to evaluate the differences; measured weights were not adjusted for dehydration status. For groups of patients aged 15 and older, we compared the distribution of measured weights to the gender-specific weight estimates with the Wilcoxon signed rank test. Measured weights during the intervention at the sub-district were unfortunately not obtained, and therefore, to assess how well the estimated weights of patients younger than 15 years of age matched the measured weights, we included district measurements and only sub-district pre-intervention measurements. For those aged 15 and older, we compared estimated weights to district intervention measurements only (since we used measured weights during pre-intervention to obtain estimated weights for these ages). After determining that the weight estimates for children less than 5 years of age were too high, we evaluated WHO percentiles and identified the percentile that best approximated weight for age in this cohort as the percentile that minimized the root mean square error (RMSE) between measured weights and gender-specific WHO percentile estimated weights. All summary statistics and statistical analyses were completed in the statistical software package R version 3.2.2 [34]. All data except for a few restricted items are provided in the supplementary materials (S3 Dataset).

## Results

### Population Characteristics

A total of 841 patients were enrolled and their records analyzed (Table 1). In the pre-intervention period, we enrolled 325 patients (204 district and 121 sub-district), and in the intervention period, we enrolled 516 patients (430 district and 86 sub-district). Onboarding periods prior to both the pre-intervention and intervention periods were permitted to accommodate for unanticipated additional training and mitigate possible Hawthorne effects (see timeline section). During these periods, 113 additional patients were enrolled but were excluded from the analysis. No patients were excluded for co-morbidities because in practice, these patients were admitted directly to the non-diarrheal wards for more advanced care prior to study screening. Seven patients (0.8%) had less than 3 loose stools in 24 hours and were excluded from the analysis (see inclusion criteria). Comparing the pre-intervention and intervention periods in the district hospital, we observed significant differences (*p* < 0.001) in the proportions of children less than 5 years and adults 20 years and older (Table 1); therefore, we controlled for age in subsequent analyses. A total of 50 doctors participated in the study with participation generally spanning study periods; 35 doctors participated at the district hospital (27 pre-intervention period; 17 intervention) and 15 doctors participated at the sub-district hospital (13 pre-intervention; at least 3 intervention)

**Table 1:** Population characteristics during the pre-intervention and intervention periods.

Population characteristics	All Patients N = 841	District			Sub-district		
		Pre-intervention N = 204	Intervention N = 430	*p*-value^a^	Pre-intervention N = 121	Intervention N = 86	*p*-value^a^
Age, years; N (%)							
0–4	381 (45.3)	39 (19.1)	240 (55.8)	**< 0.001**	57 (47.1)	45 (52.3)	0.71
5–9	27 (3.2)	9 (4.4)	7 (1.6)	0.06	8 (6.6)	3 (3.5)	0.53
10–14	26 (3.1)	5 (2.5)	14 (3.3)	0.80	4 (3.3)	3 (3.5)	1.00
15–19	36 (4.4)	10 (4.9)	19 (4.4)	0.84	5 (4.1)	2 (2.3)	0.70
≥20	371 (44.1)	141 (69.1)	150 (34.9)	**< 0.001**	47 (38.8)	33 (38.4)	1.00
Sex, Female; N (%)	403 (47.9)	113 (55.4)	200 (46.5)	0.24	45 (37.2)	45 (63.2)	0.20
Watery stool; N (%)	840 (99.9)	204 (100)	429 (99.8)	1.00	121 (100)	86 (100)	1.00
Bloody stool; N (%)	1 (0.1)	0 (0)	1 (0.2)	1.00	0 (0)	0 (0)	1.00
Stools in 24 h; N (%)							
Data not available	6 (0.7)	3 (1.5)	2 (0.5)	0.34	0	1 (1.2)	0.42
3 to 6	124 (14.7)	14 (6.9)	59 (13.7)	**0.02**	45 (37.2)	6 (7.1)	**< 0.001**
7 to 12	263 (31.3)	54 (26.5)	61 (14.2)	**0.003**	76 (62.8)	72 (84.7)	0.20
>12	448 (53.3)	133 (65.2)	308 (71.6)	0.50	0 (0.0)	7 (8.2)	**0.003**
History of^b^; N (%)							
Meds (any) and/ or ORS	326 (38.8)	61 (29.9)	64 (14.9)	**< 0.001**	115 (95)	86 (100)	0.84
Use of ORS	286 (34)	29 (14.2)	56 (13.0)	0.71	115 (95.0)	86 (100)	0.84
Ciprofloxacin	34 (4)	19 (9.3)	28 (6.5)	0.26	4 (3.3)	1 (1.2)	0.65
Azithromycin	29 (3.4)	14 (6.9)	12 (2.8)	**0.03**	3 (2.5)	0 (0.0)	0.27
Metronidazole	55 (6.5)	23 (11.3)	30 (7.0)	0.13	2 (1.7)	0 (0.0)	0.51

^a^ The *p*-values indicate whether there were significant (bold) differences between the pre-intervention and intervention; district and sub-district tested separately.

^b^ History of taking medications for this illness (e.g. diarrhea). No time-frame was specified.

### Microbiologic Findings

A total of 277 targeted cultures were performed and identified *V*. *cholerae* (N = 19; 7%); *Aeromonas* spp. (N = 19; 7%), *Shigella* spp (N = 5; 2%), and *Salmonella* spp (N = 3; 1%); note that the approach was targeted and did not test for viral pathogens and other common bacterial pathogens. *V*. *cholerae* strains were sensitive to azithromycin (19/19) and ciprofloxacin (19/19), had intermediate sensitivity to cotrimoxazole (16/19) and tetracycline (14/17), and were resistant to erythromycin (19/19). One cholera patient was co-infected with at least *Aeromonas* spp.

### Clinical Assessment and Management

#### Weight estimation

**Fig 2:**
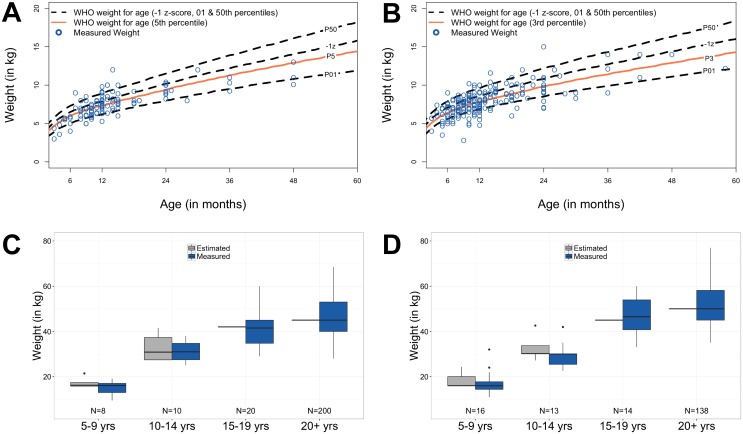
Weight estimation. (A and B) Measured weights for female (N = 120) and male patients (N = 216) less than 5 years, respectively. (C and D) Measured and estimated weights during the pre-intervention and intervention periods for older female and male patients, respectively. Data are presented as standard box plots; estimated weights used during the study are 42 kg for females 15–19 years; 45 kg for female 20+ years; 45 kg for males 15–19 years; 50 kg for males 20+ years.

Standard of care at both study hospitals relied on a ‘best guess’ weight because scales historically have not been available. During the intervention, the Rehydration Calculator provided weight estimates (see methods). For children less than 5 years of age, the sex-specific estimated weight-for-age (WHO -1 z-score) compared to weights independently measured by the study team were high for both sexes (both *p* < 0.001; Fig 2A females and Fig 2B males). The analysis revealed that measured weights for girls (N = 120) better matched the WHO 5^th^ percentile (*RMSE* = 1.17; Fig 2A) while boys’ measured weights (N = 216; Fig 2B) were lower and better matched the WHO 3^rd^ percentile (*RMSE* = 1.25). Differences between estimated and measured weights for all other age groups were not significant (Fig 2C and 2D).

#### Clinical assessment

**Fig 3:**
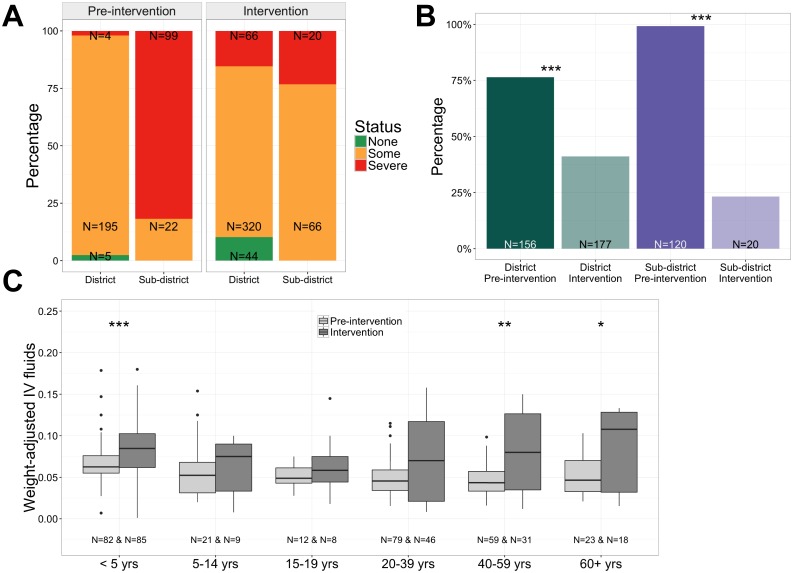
Dehydration status and IV fluid administration. (A) Percentages of patients with dehydration status ‘none’, ‘some’, or ‘severe’; IV fluids were recommended for severe dehydration (red). (B) Percentages of patients who received IV fluids. (C) Weight-adjusted IV fluid volume ordered by age group (ml ordered/patient weight in kg; the value 0.10 represents the recommended volume for severely dehydrated patients). *p<0.05, **p<0.01, ***p<0.001.

During the pre-intervention period, dehydration status was assessed by the clinician, without guidance by the Rehydration Calculator (Fig 3A), and we observed a large difference between the percentages of patients admitted with severe dehydration at the district hospital (4/204; 2%) and the sub-district hospital (99/121; 82%). However, once the Rehydration Calculator was used in the intervention period, there was no significant difference in the percentage of patients assessed as severely dehydrated between the two sites (66/430, 15% district; 20/86, 23% sub-district; *p* = 0.17).

#### Clinical management

**Fig 4:**
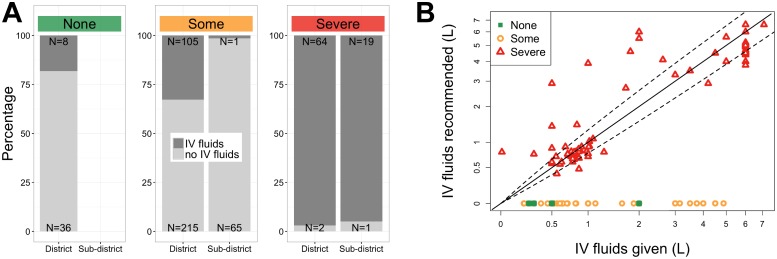
Dehydration status and IV fluid administration in the intervention period. (A) Percentages of patients in the intervention period (N = 516) for whom IV fluids were/were not ordered by dehydration status (‘none’, ‘some’, or ‘severe’). (B) Comparison of volume of IV fluid recommended versus ordered during the intervention period for those patients who received IV fluids (N = 197); diagonal line represents agreement between the volume recommended and ordered; dashed lines represent differences of 30%.

During the intervention, the proportion of patients prescribed IV fluids significantly decreased at both hospitals (both *p* < 0.001; Fig 3B). At the district, the RR was 0.5 (pre-intervention 77% and intervention 41%) and at the sub-district, the RR was 0.2 (pre-intervention 99% and intervention 23%). Although the proportion of patients prescribed IV fluids decreased, the weight-adjusted volume of IV fluids increased for children less than 5 years (*p* < 0.001), adults ages 40 to 59 (*p* = 0.003), and adults 60 and older (*p* = 0.03; Fig 3C). Considering only those patients in the intervention period for whom we had objective measures of dehydration status (Fig 4A; N = 516 total) and for whom IV fluids were ordered (Fig 4B; N = 197), we found that the IV fluid volume ordered in severely dehydrated patients was congruent (did not differ by more than 30%) with the IV fluid volume recommended (*p* = 0.1; Fig 4B). Although IV fluids are generally recommended for severely dehydrated patients, IV fluids were ordered for patients with ‘none’ and ‘some’ dehydration (Fig 4).

**Fig 5:**
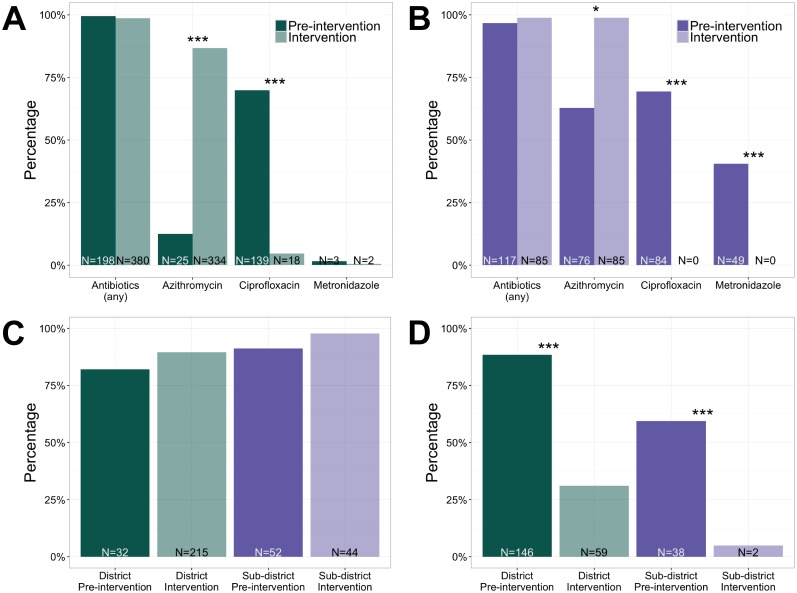
Antibiotic and zinc guideline adherence. (A and B) Percentages of patients for whom antibiotics were prescribed when azithromycin was recommended at the district and sub-district hospitals, respectively. (C) Percentages of zinc prescriptions for patients under 5 years (recommended). (D) Percentages of zinc prescriptions for patients over 5 years (not recommended). *p<0.05, **p<0.01, ***p<0.001.

Antibiotic use approached 100% during the pre-intervention period (320/325) and remained high in the intervention period (494/526); this was in the context of the majority of patients diagnosed with acute watery diarrhea with moderate or severe dehydration (Table 1). Given cholera in the study area, the recommended antibiotic for acute watery diarrhea is azithromycin. To determine antibiotic guideline adherence, we analyzed only those records in which azithromycin was recommended (199/204; 98% district pre-intervention, 385/430; 90% district intervention, 117/121; 97% sub-district pre-intervention, and 86/86; 100% sub-district intervention) and found that proportions of azithromycin prescriptions significantly increased at both hospitals (district *p* < 0.001; 13% pre-intervention to 87% intervention; sub-district *p* = 0.35; 63% pre-intervention to 99% intervention) with RRs of 6.9 and 1.6, respectively (Fig 5A and 5B). During the intervention, use of antibiotics not recommended for acute watery diarrhea in our study location (e.g. ciprofloxacin and metronidazole) significantly decreased. For ciprofloxacin, the RR was 0.1 at the district hospital (pre-intervention 70%, 5% intervention, *p* < 0.001), and ciprofloxacin use ceased entirely at the sub-district hospital (69% pre-intervention, 0% intervention, *p* < 0.001). Metronidazole was rarely used at the district hospital and use ceased entirely at the sub-district hospital (40% pre-intervention and 0% intervention; *p* < 0.001). For patients less than 5 years of age, zinc use was high and adhered well to guidelines during both periods (Fig 5C); the small increase with the use of the calculator was not significant at either hospital (*p* = 0.80 district, 82% pre-intervention and 90% intervention; *p* = 0.89 sub-district, 91% pre-intervention and 98% intervention) with RRs of 1.1 at both hospitals. Zinc is not typically indicated for patients older than 5 years of age (Fig 5D) and in this group, zinc use significantly decreased with RRs at the district hospital of 0.4 (pre-intervention 89%, intervention 31%) and at sub-district hospital of 0.1 (pre-intervention 59%, intervention 5%; both hospitals *p* < 0.001).

#### Hospital and post-discharge course

During hospital admission, primary measures included the duration of admission, the type of discharge, and complications (Table 2). At the district hospital, there was an increase in the proportion of patients with ‘discharged on request’ (DOR) during the intervention period but the duration of admission was not significantly different. A DOR is a discharge level that is permitted but not encouraged by the physician. It is not at the legal level of ‘discharged on risk-bond’ (aka discharged against medical advice). At the sub-district hospital, the duration of admission was shorter (27.9 versus 24.6 hours, *p* < 0.001) during the intervention period. The post-discharge course was evaluated at 10 days for most patients (N = 596; 71%). Differences between the pre-intervention and intervention periods included a one day reduction in post-discharge days of diarrhea at the district hospital for adults (*p* < 0.001), and a one-day increase in post-discharge days of diarrhea at the sub-district hospital for children less than 5 years and adults (*p* < 0.001 for both). Although not significantly different, there was one mortality of a young child that occurred during the intervention period at the district hospital and a verbal autopsy was performed. In brief, diarrhea and emesis were resolving on post-discharge day 1, abdominal swelling of unknown cause was noted on post-discharge day 2, the family received outpatient treatment on post-discharge day 3, and the patient decompensated and died on post-discharge day 4. The death was reported to both the IEDCR and Stanford IRBs and determined to be independent of the intervention.

**Table 2:** Hospital and post-discharge course.

Hospital course	District			Sub-district		
	Pre-intervention N = 204	Intervention N = 430	*p*-value^a^	Pre-intervention N = 121	Intervention N = 86	*p*-value^a^
Duration of admission (h); median (Q1,Q3)^b^	24.1 (17.2,34.4)	25.0 (18.3,41.7)	0.09	27.9 (20.8,39.5)	24.6 (21.9,29.6)	**< 0.001**
Complications^c^	2 (1)	1 (0.2)	0.24	0 (0)	0 (0)	-
Discharge type; N (%)						
with advice	128 (62.7)	210 (48.8)	0.07	1 (0.8)	1 (1.2)	1
on request	32 (15.7)	145 (33.7)	**< 0.001**	113 (93.4)	84 (97.7)	0.84
against medical advice	11 (5.4)	13 (3.0)	0.18	1 (0.8)	0 (0)	1
absconded	31 (15.2)	53 (12.3)	0.39	6 (5.0)	1 (1.2)	0.25
other	2 (1.0)	9 (2.1)	0.52	0 (0)	0 (0)	-
Mortality; N (%)	0 (0)	0 (0)	-	0 (0)	0 (0)	-
Post-discharge course	N = 138 (67.6%)	N = 300 (69.8%)		N = 94 (77.7%)	N = 64 (74.4%)	
Days for diarrhea to resolve; median (Q1,Q3)						
Ages 0–4	4 (3,5) N = 23	4 (3,5) N = 172	0.91	1 (1,1) N = 44	2 (1,3) N = 33	**< 0.001**
Ages 5–9	2.5 (2,3) N = 6	3 (3,4) N = 5	0.12	1 (1,1) N = 6	1.5 (1.25,1.75) N = 2	0.66
Ages 10–14	4.5 (3.75,5.25) N = 2	2.5 (2,3) N = 10	0.13	1 (1,1) N = 2	1.5 (1.25,1.75) N = 2	0.62
Ages 15–19	4 (2.75,4) N = 8	3 (2,5) N = 18	0.86	1 (1,1.25) N = 4	3 (3,3) N = 2	0.08
Ages ≥20	4 (3,5) N = 99	3 (2,4) N = 95	**< 0.001**	1 (1,1) N = 38	2 (1,2) N = 25	**< 0.001**
Readmitted; N (%)	4 (2.9%)	2 (0.7)	0.09	0 (0)	0 (0)	1
Mortality; N (%)	0 (0)	1 (0.3)	1	0 (0)	0 (0)	-

^a^ The *p*-values indicate whether there are significant (bold) differences between pre-intervention and intervention; district and sub-district tested separately.

^b^ Q1,Q3 are the 1st and 3rd quartiles. N = 187 & 417 for district pre-intervention and intervention; N = 112 & 86 for sub-district pre-intervention and intervention.

^c^ Complications were classified as respiratory, with a sub-classification for fluid overload, and other.

## Discussion

The objective of this clinical pilot study was to evaluate the impact of a smartphone adaptation of the World Health Organization (WHO) diarrheal disease management guidelines. The calculator was associated with significant and positive prescription change towards guideline adherence. The findings support our hypothesis that technology-enabled decision-support tools can promote evidenced-based practice in resource-limited settings.

With respect to basic medical care, all medical facilities should have a functional and suitable weight scale. However, this minimal standard is frequently untenable in resource-limited settings like those in this study, and therefore, clinicians are forced to estimate weight. We designed the calculator to estimate pediatric weight for age by -1 z-score during the study; however, post-study analysis showed measured weights approximated best to the 5^th^ (females) and 3^rd^ (males) WHO percentiles for children less than 5 years of age while estimates for older age groups did not differ significantly from measured weights. These low percentiles in children under 5 years are not likely to be due to the degree of dehydration at admission alone, but suggest that admitted patients in this age range may be a particularly vulnerable subset of the population. Future larger studies are required to expand on these findings. Additional anthropomorphic measurements (e.g. mid-upper-arm circumference length; MUAC) may also provide evidence that decision-support tools must be built to leverage locally available data in order to optimize weight and other anthropomorphic estimations. In the preparatory phase, we experienced surprising resistance to the use of MUAC bands for weight estimation or for malnutrition assessment [35, 36] because of past experience with the time required to take an accurate MUAC and difficulty maintaining MUAC strip supplies.

Rehydration Calculator deployment was associated with a reduction in overall IV fluid prescriptions but an increase in the volume ordered when IV fluids were prescribed. Previous studies have shown that transitioning low and medium risk patients towards oral rehydration solution reduces the risk of complications, provides cost-savings benefit to both patients and institutions, and remains effective [37]. The weight measurements enabled the calculator to provide weight-based dosing of fluids for the pediatric patients. The adult patients were often dosed with categorical fluid volumes (e.g. 1, 2, and 3 liters) which may be more practical for staff. In the intervention period, the fluid volumes ordered versus recommended for severely dehydrated patients during the intervention were congruent. In a setting like Netrokona District that treats 5000 diarrheal patients monthly, a safe decrease in IV fluid use represents significant overall cost-savings to the medical system while empowering clinicians with the ability to increase fluids volumes to the recommended dose when IV fluids are indicated. Therefore in a venue like Netrokona with low diarrhea-associated mortality, a safe reduction in IV fluid orders may be an important advantage of decision-support tools like the Rehydration Calculator. Future studies in areas with high morbidity and mortality are required to evaluate the physiologic benefits.

The Rehydration Calculator was associated with significant and positive antibiotic class-switching to the recommended agent azithromycin. Almost all patients were described as having acute watery diarrhea with moderate or severe dehydration. For these reasons, azithromycin was recommended for almost all patients out of concern for cholera. However, 7% of samples cultured were positive for *V*. *cholerae* [all tested strains were azithromycin-sensitive]. The presumed remaining 93% of patients with non-bloody (e.g. non-Shigellosis, non-Salmonellosis, non-Campylobacter) and non-cholera diarrhea likely did not require antibiotics. Although the calculator provided objective dehydration classification, we must also encourage clinicians to accurately report ‘acute watery diarrhea’ and deploy point-of-care diagnostic surveillance for *V*. *cholerae* to decrease the prescription of antibiotics. Although there was a small increase (1 day) in the duration of post-discharge days of diarrhea at the sub-district hospital, a larger study will be required to support this finding and determine if this finding has clinical significance. More importantly, this study should spark discussion on the utility of using the phrase “acute watery diarrhea” given that casual use may have contributed to an overuse of antibiotics. A reasonable alternative, despite some culture and physiologic nuance, would be ‘rice-water stool’ because this phrase implies that the clinician highly suspects cholera.

The first goal of this study was to improve adherence to WHO-derived guidelines that are evidence-based and considered the gold standard for safety and effectiveness. Although we were only reformatting these guidelines to a smartphone medium, we monitored secondary safety related outcomes, and found only minor differences in length of stay and type of discharge. At the district hospital during the intervention, there was one post-discharge death that occurred after the majority of the diarrheal symptoms had resolved (see hospital and post-discharge course). This event generated a case fatality rate (CFR) of 0.1% for the entire study (1 out of 954 total patients including patients in the onboarding periods) and 0.3% for the district intervention which are similar to expected CFRs at similar sites [17, 19]. These preliminary data suggest that the Rehydration Calculator is a safe tool to improve guideline adherence for diarrheal disease decision-support. Future studies that include more detailed physiologic assessments may provide further avenues to assess safety and support these initial findings.

A second goal was to develop the Rehydration Calculator to be scalable and desired by medical professionals in hospitals like those in Netrokona. Frameworks are being built to guide development and evaluation of smartphone decision-support tools [38]. Scalability of the Rehydration Calculator will likely depend on the use of personal smartphones. This is feasible because the Rehydration Calculator has minimal to no associated cost, captures no personal health information, and does not rely on connectivity. Venues for Rehydration Calculator deployment are broad. For example, Bangladesh and Pakistan both have considerable diarrheal disease, including cholera, and rank among the top ten globally for cell phone penetration per capita [39]. Professionals in these countries are rapidly transitioning to affordable Android smartphones. It also remains possible for institutions to embrace the Rehydration Calculator. This would require purchasing dedicated phones, training, and technical support to maintain the phones. Future studies will require rigorous qualitative and quantitative analysis to further these goals of scalability for both individual and/or institutional deployment.

Scalability also relies on desirability. Although an anonymous user acceptance survey was not performed, feedback was obtained in a group format to assess end-user experience. Clinicians expressed that the calculator expedited both clinical assessment and reduced the time required to generate a treatment plan (3–5 minutes). Additionally, use of the calculator conferred a level of professional prestige that was attractive. Physicians were allowed to deviate from the guidelines as needed. Clinicians vocalized that deviation was important when patients expected IV fluids or when emesis made oral rehydration inappropriate. These experiences likely manifested in IV fluid orders for patients with some or no dehydration (Fig 4A). Despite the lack of user acceptance surveys, the design of the Rehydration Calculator appeared to match clinician needs. Future studies are required to explore these preliminary observations.

The findings in this study should be viewed within the context of the limitations of the study design and available data. The study site is likely generalizable to remote rural settings with a significant burden of water-borne disease. However, the remoteness also made exhaustive oversight difficult and contributed to the limitations: (i) The first limitation was a suspected under-enrollment of patients at the district hospital during the pre-intervention period compared to intervention period. This suspected under-enrollment may have contributed to the age difference between the pre-intervention and intervention periods; we therefore accounted for age in the analysis. (ii) The second limitation occurred during the intervention period at the sub-district hospital where weight measurements were not obtained due to a work-flow issue. Thus, when evaluating the accuracy of weight estimates, we excluded sub-district patients enrolled during the intervention period. (iii) There was no independent assessment of dehydration during the pre-intervention phase, medications and fluids prescribed were not confirmed to have been administered, and objective physiologic outcome measures were not reliably obtained. The data collection method also relied heavily on the physician transcribing the assessment and plan to the paper chart, and then the researcher transcribing this information to the digital data collection platform. Future studies need a tractable method to validate accuracy through this chain of events; independent assessment would provide added quality control for these steps. (iv) The study was designed as a pilot study, and although the results from the two sites were similar, there was no concurrent control group; this puts the study at risk of temporal variation during the 12-week study. (v) Exclusion criteria were unbalanced because severely malnourished patients were excluded during the intervention because of limited clinical capacity, however, this limitation actually had no impact on the results because no patients were excluded for severe malnutrition alone. (vi) The study was not designed to perform sub-analysis at the level of individual clinicians because of logistical limitations and sensitivity concerns amongst stakeholders. Despite these limitations, the core results from this study were robust and can be viewed as a critical step towards improving the quality of care in remote regions inflicted with diarrheal disease. These findings warrant further investigation with a cluster randomized controlled trial (cRCT). One approach to address physician gestalt and make the cRCT more generalizable and standardized would be to randomize the control study arm to use a paper-based version of WHO guidelines via a laminated card and the intervention arm to the WHO guidelines on the Rehydration Calculator.

In conclusion, this pilot study highlights that significant improvement towards guideline adherence can be achieved when clinicians are provided a tool such as the Rehydration Calculator. The study also demonstrates how a simple tool can standardize the assessment of dehydration status, which is a critical first step to understanding physiologic determinants of severe disease and identifying opportunities to improve clinical acumen. Upon further investigation in a cRCT, we hope that tools like the Rehydration Calculator in the hands of frontline providers will contribute to the ongoing drop in morbidity and mortality from diarrheal disease as well as usher in a new era of clinical approach and scientific understanding.

## Supporting Information

S1 Movie Rehydration calculator overview movie. Overview movie of the version of the Rehydration Calculator deployed in the pilot study. Access at http://purl.stanford.edu/sv492bk0032.
(MOV)

S1 Software Rehydration calculator software. The most recent version of the software can be downloaded directly from the google playstore by searching for “Outbreak Responder” or directly at https://play.google.com/store/apps/developer?id=Outbreak+Responder&hl=en. Performance is best on a Samsung Android device with a 5 inch screen; compatibility does vary between companies and Android models. At the time of publication, the only software update from the version in the pilot study was a change to estimate weight for age in Bangladesh (5^th^ percentile and 3^rd^ percentile for females and males, respectively). Future changes will be documented in the information tabs from within the software platform.
Disclaimer: Access to the this software is provided for academic evaluation only. While the software remains under clinical study, the prototype is not intended for clinical practice. Accordingly, the software is password protected. After downloading the software, please contact the corresponding author Dr. Eric Nelson, MD PhD at eric.nelson@ufl.edu or outbreakresponder@gmail.com for the application key. Login credentials for the data collection module can also be provided upon request.
(DOCX)

S1 Dataset All data except for a few restricted items to protect the identification of patients are provided in the dataset. In the de-identified data sheet, the single mortality was removed, the study location name and measured weight for children less than 15 years was removed, the ages for the two patients older than 89 years were grouped as “greater.than.89” for age in years and “greater.than.1068” for age in months, and a calculated hospital admission duration was provided (e.g. not based on dates). The ID number was randomly generated and assigned. The de-identified data sheet for the weight of patients less than 15 years old contains only measured weights (no measured weights were available in the intervention period in Madan), estimated weights, gender and age.
(XLSX)
