# Supplementary material for: Evaluation of a Smartphone Decision-Support Tool for Diarrheal Disease Management in a Resource-Limited Setting
Source: PLoS Negl Trop Dis. 2017 Jan 19;11(1):e0005290. doi: 10.1371/journal.pntd.0005290 (PMC5283765; doi:10.1371/journal.pntd.0005290)
Supplement: S1 Software — The most recent version of the software can be downloaded directly from the google playstore by searching for “Outbreak Responder” or directly at https://play.google.com/store/apps/developer?id=Outbreak+Responder&hl=en. Performance is best on a Samsung Android device with a 5 inch screen; compatibility does vary between companies and Android models. At the time of publication, the only software update from the version in the pilot study was a change to estimate weight for age in Bangladesh (5th percentile and 3rd percentile for females and males, respectively). Future changes will be documented in the information tabs from within the software platform. Disclaimer: Access to the this software is provided for academic evaluation only. While the software remains under clinical study, the prototype is not intended for clinical practice. Accordingly, the software is password protected. After downloading the software, please contact the corresponding author Dr. Eric Nelson, MD PhD at eric.nelson@ufl.edu or outbreakresponder@gmail.com for the application key. Login credentials for the data collection module can also be provided upon request. (DOCX) [file pntd.0005290.s002.docx]

The most recent version of the software can be downloaded directly from the google playstore by searching for “Outbreak Responder” or directly at <https://play.google.com/store/apps/developer?id=Outbreak+Responder&hl=en>
